# Conscience in health care and the definitions of death

**DOI:** 10.3325/cmj.2013.54.75

**Published:** 2013-02

**Authors:** Yutaka Kato

**Affiliations:** Department of Bioethics, Graduate School of Medicine, Dentistry, and Pharmaceutical Sciences, Okayama University, Okayama, Japan

## Abstract

Brain death or neurologic death has gradually become recognized as human death over the past decades worldwide. Nevertheless, in Japan, the New York State, and the State of New Jersey, one can be exempt from death determination based on neurologic criteria even in the state of brain death. In Japan, the 1997 Act on Organ Transplantation legalized brain death determination exclusively when organs were to be procured from brain-dead patients. Even after the 2009 revision, the default definition of death continued to be cardio-pulmonary criteria, despite the criticism. The cases of Japan and the United States provide a good reference as social experiments of appreciating conscientious or religio-cultural dimensions in health care. This text theoretically examines the 1997 Act on Organ Transplantation of Japan and its 2009 revision, presenting some characteristics of Japan’s case compared to American cases and the implications its approach has for the rest of the world. This is an example in which a foreign idea that did not receive widespread support from Japanese citizens was transformed to fit the religio-cultural landscape.

Brain death (neurologic death) has gradually been recognized as human death over the past decades worldwide. Nevertheless, in Japan, the New York State, and the State of New Jersey, one can be exempt from death determination based on neurologic criteria even in the case of brain death. The New York State established the Guidelines for the Determination of Brain Death (1987, 1995, 2005) to accommodate religious or moral objections to brain death (1). The State of New Jersey also enacted the Declaration of Death Act (1991) to accommodate religious objections to brain death (2). All this resulted from the accommodation of religious and moral objections to neurologic criteria. Hans-Martin Sass argued for “a formula for a global Uniform Determination of Death statute, based on the ‘entire brain including brain stem’ criteria as a default position, but allowing competent adults by means of advance directives to choose other criteria for determining death during the process of dying (3).” These cases provide a good reference as social experiments in order to evaluate this formula.

In the text, the term “conscience” or its adjective form is chosen as a superordinate concept to moral/religious belief according to conventional usage. Conscience might appear universal whereas religio-cultural dimension differs among nations. In this text, conscience is considered to manifest itself within different societal traditions.

## The 1997 Act on Organ Transplantation of Japan and the 2009 revision

In Japan, following a decade-long national debate on brain death and organ transplantation, the 1997 Act on Organ Transplantation legalized brain death determination exclusively when organs are to be procured from a brain-dead patient. Antipathy to brain death determination among the Japanese was often regarded as the primary reason for this prolonged law-making process. Japan’s case was a prototypical example in which health care was influenced by religio-cultural differences.

Under the 1997 Act on Organ Transplantation, brain death as a criterion for death determination was applied only to those who had consented to donate their organs and whose family did not refuse organ procurement after determination of brain death. Otherwise, death determination was performed on cardio-pulmonary (triad of death) criteria, which did not determine brain death and which was the default death definition. Consent by the patient himself/herself to donate organs in the brain death state was considered indispensable. This provision was a result of compromises in the Diet (Japanese Parliament). The resulting shortage of transplantable organs was ascribed to the restrictive provisions of this act. The first revision was expected in 3 years but it was substantially delayed.

The delayed revision in 2009 went into effect the following year (4). Some criticized the original draft for the revision for trying to adopt an alternative definition. Although the double standard scheme within the 1997 act was considered as transient, the death determination based on brain death criteria remained exclusively limited to organ donors. The default definition of death continued to be cardio-pulmonary criteria.

The revision enabled organ procurement/transplant for children, and it enabled organ procurement from patients whose will was unknown, as long as the family consented. Not only the patients themselves but also their families were entitled to refuse brain death determination. If the patient was willing to donate organs but the family refused, the patient’s will was overridden. In these cases, the families’ objection can overturn the patients’ acceptance of brain death. Significantly, unless otherwise instructed, the patient was assumed to be willing to donate organs. But such assumption was disguised as familial or surrogate decision-making, which appeared more acceptable as well as familiar in Japanese society.

## Disputable roles of conscience

There is no consensus regarding the influence of religiosity or conscience on Japan’s case. Scholars, including Robert Veatch (5,6) and Canadian medical anthropologist Margaret Lock (7), have ascribed Japan’s adherence to cardio-pulmonary death definition to religio-cultural factors (5). Veatch noted that “It is possible that the uniquely Japanese resistance to brain-based definitions of death should be traced more to indigenous religious beliefs called Shintoism (6).” Some scholars have pointed out the significance of the body in perceiving death and interpersonal dimension of death (meaning that it is important for families to look at the body and understand the person is dead). Dissenters, who were against brain death determination in the early 1990s, in the Provisional Commission for Study on Brain Death and Organ Transplantation (Japan) relied on the uniquely Japanese religio-cultural background (8-10).

But some are skeptical about the significance of religiosity. Japanese society today has the custom of cremation, which appears contradictory to appreciation of the body. According to some surveys, the majority of Japanese was for brain death definition as early as the 1980s (11). The ratio of those who were for the neurological definition of death was not very different from those of other countries. Furthermore, resistance to brain death determination was not only religiously-motivated; doubt on the scientific validity of assessment procedures, and mistrust in the health care service (evoked by Wada case in 1968) contributed as well. Additionally, Shinto and the indigenized form of Buddhism give few clear instructions on health care. Therefore, it was difficult to develop a convincing argument based on religious writings.

Nevertheless, death is an exceptionally frequent subject in Shinto and Buddhism traditions, though these religions do not have a systematic way of actualizing their teachings. Importantly, during the law-making process, parties (except the Communist Party) allowed their members to vote according to their individual standpoints (very rare). Their political views and positions were less significant in their voting decisions (12). In addition, religions in Japan are Shintoism, with 108 427 100 members (85.0% of the national population), Buddhism with 87 506 504 members (68.6%), and Christianity with 2 369 484 (1.86%) members, and others (the total population is 127.51) (13). In the meantime, approximately 70% of Japanese in some surveys answered that they were “non-religious.” Their self-understanding as "non-religious," despite their often plural affiliation, is apparently based on a narrower definition of religiousness as conscious adherence to an established/revealed religion (differing from the Japanese indigenous religious tradition) (14). In Japan’s case, definitions of religions or religiousness should be broader and inclusive. Moreover in Japan, where the Medical Practitioners Act stipulates the duty to rescue (Article 19), health care mostly lacks the practice of conscientious objection both by patients and health care professionals. Few Japanese health care professionals are religiously motivated in their vocation, primarily because the Japanese religious traditions have been scarcely interested in health care. The vast majority of Japanese health care professionals were in favor of the brain death determination (15). Therefore, the primary objection to brain death determination was not scientific. Obviously conscientious objection influenced the law-making process in the form of religio-cultural influence.

## The implications of Japan’s approach

After the act was revised, the number of organ donations did not increase (the total number of donations after brain death and cardio-pulmonary death remained roughly the same), which made some think there was an urgent necessity to alter the default death definition. Thus, a further revision is likely. Still the current Japan’s organ transplant act is a typical case of internationally exceptional legislation and it can serve as a unique and significant reference.

First, the double-standard death definitions did not cause serious social disruption, except for a shortage of transplantable organs. With this scheme, in Japan, brain death determination need not be uniformly implemented, but allowed as an exception, even when a certain percentage of the population come to prefer brain death determination, and in order to protect the values held by the minority. Although the enactment and the revision at first aimed at consensus on uniformity in death determination and failed, the resulting consensus for accommodation of the double standard death definitions is more beneficial than harmful.

The New Jersey and New York cases have endorsed different levels of conscience (religious and religious/moral reasons, respectively). Japan’s case has been influenced by religiosity or conscience but the organ transplant act of Japan grants no privileged/special status to conscience. Japan’s approach has integrated the religio-cultural dimension into the default procedure, thus exempting health care professionals from judging conscience individually and exempting the nation internationally. There remains a question on the justifiability of the privileged status of conscience. Conscience apparently has distinct qualities, namely consistency, sincerity, intensity, and so forth, which can elicit special consideration and mean greater accountability. However, it is at the political discretion of each nation whether it grants conscience a privileged status. In the United States, they have constitutional foundations. As long as they are conscientious, the free exercise clause in the First Amendment to the US Constitution is applicable. Veatch argued that “The principle of equal respect would seem to require that if religious objections were permitted, equally sincere, and equally deeply held, nonreligious philosophical objections would be equally acceptable.” He argued that exemption can be extended to comparable objections based on precedential court decisions on conscientious objection in military service and employment (16). Japan has a comparable constitutional stipulation – Article 20 (The Constitution of Japan) explicitly stipulates freedom of religion. This constitutional stipulation apparently grants a privileged status to conscience. Special status of conscience is defendable in policy-making in Japan.

Finally, accommodation of conscience, which in Japan arose spontaneously through a process of lengthy debate and deliberation, can have implications for other comparable procedures in health care. In similar medical practices possibly implemented in the future, the above idea of consensus for accommodation will be a promising option, allowing conscientious objectors to be exempt from the default procedures.
